# Intrathecal triamcinolone acetonide exerts anti-inflammatory effects on Lewis rat experimental autoimmune neuritis and direct anti-oxidative effects on Schwann cells

**DOI:** 10.1186/s12974-019-1445-0

**Published:** 2019-03-09

**Authors:** Kalliopi Pitarokoili, Melissa Sgodzai, Thomas Grüter, Hussein Bachir, Jeremias Motte, Björn Ambrosius, Xiomara Pedreiturria, Min-Suk Yoon, Ralf Gold

**Affiliations:** Department of Neurology, St. Josef Hospital, Ruhr-University Bochum, Gudrunstr. 56, 44791 Bochum, Germany

**Keywords:** Autoimmune neuropathies, Triamcinolone, Experimental autoimmune neuritis, Autoimmunity, Inflammation, Immunotherapy

## Abstract

**Background:**

Corticosteroids dominate in the treatment of chronic autoimmune neuropathies although long-term use is characterized by devastating side effects.

**Methods:**

We introduce the intrathecal application of the synthetic steroid triamcinolone (TRIAM) as a novel therapeutic option in experimental autoimmune neuritis in Lewis rats

**Results:**

After immunization with neuritogenic P2 peptide, we show a dose-dependent therapeutic effect of one intrathecal injection of 0.3 or 0.6 mg/kg TRIAM on clinical and electrophysiological parameters of neuritis with a lower degree of inflammatory infiltrates (T cells and macrophages) and demyelination in the sciatic nerve. In vitro studies in Schwann cell cultures showed an increased expression of IL-1 receptor antagonist and reduced expression of Toll-like receptor 4 after incubation with TRIAM as well as a protective effect of TRIAM against oxidative stress after H_2_O_2_ exposure.

**Conclusion:**

Intrathecal TRIAM application could be a novel immunomodulatory and potentially neuroprotective option for autoimmune neuropathies with a direct effect on Schwann cells.

## Introduction

Triamcinolone acetonide (TRIAM) is an intermediate-acting synthetic glucocorticoid, which as a sustained-release crystal suspension is suitable for sub-lesional injections for isolated psoriasis or discoid lupus erythematosus, intra-articular injections for the treatment of chronic inflammatory joint diseases, and intravitreal applications as it reduces macular edema and angiogenesis in eyes with artificial lenses [1, 2].

Regarding autoimmune diseases of the central nervous system (CNS), repeated intrathecal application of the sustained release TRIAM (40–80 mg every other day for one to three injections) is performed since the 1970s in specialized centers in Germany for primary and secondary progressive multiple sclerosis patients and improves, according to observational trials in up to 161 patients, the maximum walking distance, spasticity, and occupational deficits of the upper extremities [3–9]. Three months after repeated application of TRIAM in the cerebrospinal fluid (CSF), an elevated steroid level could still be found. Although data from large-scale clinical trials are missing, intrathecal TRIAM application is still used from specialized centers as an off-label treatment [4].

Our group has previously shown that intrathecal therapeutic application of human immunoglobulins in the context of experimental autoimmune neuritis (EAN), the animal model of dysimmune neuropathies such as Guillain-Barré syndrome (GBS), or the chronic inflammatory demyelinating polyradiculoneuropathy (CIDP) achieves a dose-dependent improvement of clinical signs, which correlate with a histological reduction of inflammatory infiltrates in the sciatic nerves and of complement activation in the sciatic nerve [10].

Corticosteroids are used as first-line therapy in the form of pulsed intravenous methylprednisolone or oral long-term prednisolone treatment for patients with CIDP and improve sensory symptoms and painful paresthesia. Unfortunately, their therapeutic benefits are limited by side effects such as osteoporosis, abdominal obesity, glaucoma, diabetes mellitus, and hypertension (Cushing’s syndrome) [2].

Intrathecal application route has not yet been investigated for TRIAM in autoimmune diseases of the peripheral nervous system (PNS) although the first sites of inflammation and increase of blood-nerve barrier permeability are the proximal nerve roots, as indicated by the early intrathecal protein increase found in these patients [11, 12].

According to the published literature, TRIAM mediates a combination of anti-inflammatory, anti-oedematous, anti-proliferative, anti-angiogenetic, and neuroprotective effects, which could be very crucial for autoimmune diseases of the PNS. The anti-inflammatory effects of TRIAM have been widely investigated and are mediated after binding to the glucocorticoid (GR) nuclear receptors, which is widely expressed in neurons and Schwann cells. Upon binding TRIAM, the receptors undergo a conformational change and translocate in the nucleus to mediate gene transcription and induce an anti-inflammatory potential for example through inhibiting cytokine release (e.g., TNFα and IL-1β). GR can also signal through nongenomic pathways which occur rapidly and do not require transcriptional changes [13, 14].

Intrathecal TRIAM was also suggested to have neuroprotective efficacy on infarction volume in acute focal cerebral ischemia in rats. Compared with controls (18.2%), infarction volume was significantly reduced using TRIAM injection into the cisterna magna (13.4%) [15].

In this study, we describe for the first time the immunomodulatory effects of triamcinolone in vivo applied intrathecally in the Lewis rat model of EAN. Moreover, this study describes indirect neuroprotective mechanisms in vitro in the context of Schwann cell culture. Therefore, we present the first report of an effective and dose-saving route of administration of TRIAM for dysimmune neuropathies.

## Materials and methods

### Antigens

The neuritogenic P2 peptide, corresponding to amino acids 53–78 of rat myelin P2 protein, was synthesized by Dr. Rudolf Volkmer from Charité Universitätsmedizin (Berlin, Germany).

### Triamcinolone

Triamcinolone acetonide (Volon A, Bristol-Meyers Squibb, New York, NY, USA, active ingredient 16a.17-Dimethyl- methylendioxy-9-fluor-11b.21-dihydroxy-1.4-pregnadien- 3.20-dion) supplied in sodium chloride solution equivalent to 10 mg TRIAM/ml or 40 mg TRIAM/ml was used for all experiments.

### Study design—disease induction and clinical score assessment

A total of 45 female Lewis rats were randomized for the therapeutic concept as described in the following section. The rats were 6–8 weeks old; they were purchased from Charles River Co. (Sulzfeld, Germany) and weighed 160–180 g when used for the following experiments. They were anesthetized by 1.5–2.0% halothane in oxygen. They were immunized by subcutaneous injection of 250 μg P2_53–78_ peptide in phosphate-buffered saline (PBS) into the root of the tail, emulsified in an equal volume of complete Freund’s adjuvant (CFA) containing *Mycobacterium tuberculosis* (1 mg/ml) H37RA (Difco, Detroit, MI, USA). Animals were weighted and scored for disease severity daily by two independent, blinded investigators. Disease severity was assessed clinically employing a scale ranging from 0 to 10 originally described by Enders et al. [16]: 0, normal; 1, less lively; 2, impaired righting/limb tail; 3, absent righting; 4, atactic gait, abnormal position; 5, mild paraparesis; 6, moderate paraparesis; 7, severe paraplegia; 8, tetraparesis; 9, moribund; 10, death. All experiments were reviewed and approved by the North-Rhine-Westphalia, Germany authorities for animal experimentation (TVA 84-02.04.2017-A023).

### Intrathecal treatment with triamcinolone and dosage rationale

The animals were anesthetized by 1.5–2.0% halothane in oxygen, and triamcinolone was injected intrathecally slowly within 4 s with a microsyringe in the following concentrations: 0.3 mg/kg, 0.6 mg/kg once (*n* = 5/group) on day 11 post-immunization (p.i.) in a volume of 5 μl, as control NaCl 0.9% in a volume of 5 μl was used (*n* = 5). The injection was performed with a 30-G needle into the four to five lumbar intervertebral space, and the correct placement of the injection was confirmed by a movement of the tail (“tail flick”) as described by Fairbanks and colleagues [17]. The experiments were repeated twice.

The dosages described above were calculated according to previous experiments from Goericke et al. who showed a reduction of the volume of infarction after an injection of 0.0012–0.3 mg/kg TRIAM in the cisterna magna in a rat model of cerebral infarction [15].

### Electrophysiological analysis

Nerve conduction tests were performed by a blinded investigator on the day before immunization (− 1) and on day 18 (maximum of natural disease course) p.i. The rats were anesthetized intraperitoneally (i.p.) with xylazine and ketamine (10 mg/kg and 50 mg/kg respectively). Using a fully digital recording Keypoint apparatus (Dantec, Skovlunde, Denmark) and paired needle electrodes inserted into the sciatic notch (hip, proximal) or the popliteal fossa (distal), the sciatic nerve was stimulated with supramaximal rectangular pulses of 0.05 ms duration and the resulting compound muscle action potential (CMAP) was recorded from needle electrodes placed subcutaneously over the dorsal foot muscles. A ground electrode was placed between the distal stimulating electrode and the active recording electrode. To calculate the motor nerve conduction velocity (MNCV), the distance between stimulating cathodes was divided by the difference of the latency. Similarly, the persistence and minimum latency of 10 F-waves evoked by stimulation at the popliteal fossa were recorded for the right side [18, 19]. Temperature differences were minimized by conducting the study as soon as the anesthesia had taken effect and by warming the leg with a heating lamp.

### Schwann cell culture and immunocytochemistry

Sciatic nerves from adult male Sprague Dawley rats (4 weeks old) were used as the source for Schwann cells (SCs), which were isolated and purified using established protocols [20]. For each culture, five animals were sacrificed by decapitation, their sciatic nerves collected and placed in Leibovitz’s L-15 medium enriched with 50 μg/ml Gentamycin (Invitrogen). Nerves were stripped of epineurium and sectioned into 1–2 mm pieces. Explants were dissociated for 18 h (37 °C, 5% CO_2_) with 1.25 U/ml dispase II (0.25%) (Boehringer Mannheim Biochemicals) and 0.05% type I collagenase (Sigma) in DMEM with 50 μg/ml Gentamycin. Dissociation was stopped with HBSS containing 40% fetal bovine serum (FBS) (Sigma), suspended and filtered through a 100-μm strainer. SC cultures were expanded overnight on poly-l-lysine (Sigma) and 1 μg/cm^2^ laminin (Sigma) coated dishes in D-10 media (Dulbecco’s modified Eagle’s medium (DMEM) (Sigma) with 10% FBS) containing 50 μg/ml Gentamycin. For a rapid expansion of the SC population, the culture medium was supplemented with a combination of 10 nM neuregulin (PeproTech) and 2 μM forskolin (Sigma) as early as 1 day after plating. To maintain a low rate of fibroblast contamination, we used Miltenyi’s MACS technology to achieve efficient separation of SCs and Thy-1-positive fibroblasts (negative selection) as described by the manufacturer’s protocol. Then, the purified SCs (purification of 90% SOX10-positive SC in flow cytometric analyses and immunocytochemistry) were kept in culture for further experiments (FACS analyses and RT-PCR analyses).

For immunocytochemical analyses, Schwann cells were seeded and incubated on poly-l-lysine- and laminin-coated coverslips for 2 days. After fixation with 4% PFA, permeabilization with 0.1% PBS-Triton, and blocking with 10% normal serum, the cells were exposed to the primary antibody S-100 (Merck, MAB079-1, mouse-anti-rat, 1:500) or SOX10 (Abcam, ab155279, rabbit-anti-rat, 1:1000). Immunoreaction was detected with the secondary antibody, goat-anti-mouse IgG-Alexa Flour 488 (1:1000, Thermo Fisher Scientific), or goat-anti-rabbit IgG-Alexa Flour 488 (1:1000, Thermo Fisher Scientific), respectively. Furthermore, nuclei were counterstained with DAPI (4′,6′-diamidino-2-phenylindole·2HCl, Biozol, Eching, Germany). The omission of the primary antibodies served as negative control. Specificity of the staining was also controlled on sections of fibroblasts.

### Histopathological assessment and immunohistochemistry

After transcardial perfusion with PBS (Gibco) on day 18 p.i. (and day 23 p.i. for a FluoroMyelin staining) we dissected the two sciatic nerves, embedded their segments in Neg-50 (Thermo Fisher, Schwerte, Germany) and snap-frozen in liquid nitrogen. We used sections of rat tissue (8 μm) on a cryostat (Leica Biosystems) mounted on glass slides (Hartenstein, Würzburg, Germany) for histopathology assessment.

For immunohistochemical staining, we used tissue sectioned in the cryostat and fixated in acetone at − 20 °C for 10 min and then exposed to the mouse monoclonal antibodies (mAb) anti-rat 15-6A1 (Hycultec, Pan T Cells CD3 1:100) and anti-rat ED1 (Hycultec, anti-CD68, macrophages, 1:100) using the avidin-biotin technique (Dako ARK KIT for Mouse Primary AB). We omitted the primary antibodies on the negative controls. Peripheral lymphoid organs served as a control for the specificity of the staining. Using 12 sections per animal and a × 40 magnification, we counted the number of positive cells. We present the results as the average cells per square millimeter tissue section.

For immunofluorescence, we incubated the sections after fixation with monoclonal antibodies (mAb) anti-ICAM-1 (1:100, Abcam, ab127160) and neurofilament H (clone N52, Abcam, 1:200), followed by incubation with secondary antibodies conjugated with Alexa 555 (1:1000) or Alexa 488 (1:1000) (Thermo Fisher, Schwerte, Germany) used according to the manufacturer’s protocol. We imbedded the slides with Fluoromount containing 4′,6′-diamidino-2-phenylindole·2HCl (DAPI) (Biozol, Eching, Germany) for fluorescent staining of DNA. For picturing the tissue, we used an inverted fluorescence microscope (BX51; Olympus, Tokyo, Japan) equipped with an Olympus DP50 digital camera.

We identified the demyelination through the accumulation of nuclei and absence of FluoroMyelin™ Red fluorescent stain (1:300, Invitrogen, Germany) performed according to the manufacturer’s protocol. For the statistic, we used the images (× 20 magnification) of 12 transverse sections of the sciatic nerve from each animal (Cell F 5.1, Olympus, Tokyo, Japan) and determined the percentage of the area of demyelination per section using image analysis software (ImageJ, National Institutes of Health, Bethesda, USA).

For histopathological assessment and immunohistochemistry, slides were blinded by a not-involved third person and labeled with a numeric code, which was unblinded after analysis.

### Isolation of mononuclear cells from lymph nodes and FACS analyses

The inguinal lymph nodes were removed after transcardial perfusion with PBS (Gibco) on day 18 p.i. under aseptic conditions. Single cell suspensions of mononuclear cells from individual rats were prepared separately (*n* = 5/group). We evaluated the frequency of CD4^+^ T cells, CD11b^+^ cells, CD4^+^CD11b^+^ dendritic cells (DCs), and CD4^+^ CD25^+^ FoxP3^+^ regulatory T cells (Tregs) by fluorescence-activated cell sorting (FACS) staining (eBioscience, San Diego, CA). FACS analyses were performed with a FACS Canto II (BD Pharmingen, Heidelberg, Germany) machine and FlowJo software (Tree Star, Ashland, Oregon). Monoclonal antibodies purchased from BD Pharmingen or eBioscience were used to detect CD4-FITC (1:500), CD11b-PE (1:200), CD25-APC (1:100), and MHC-II-Alexa647 (1:300) in accordance with the manufacturer’s instructions. Intracellular staining for Foxp3 was performed using the Foxp3 Staining Set (eBioscience) according to the manufacturer’s instructions.

To examine the protective role of TRIAM against oxidative stress, we incubated SCs for 24 h with 0.03 mM H_2_O_2_ with and without 10 μg/ml TRIAM. Cell survival was analyzed with flow cytometry (propidium iodide staining, Thermo Fisher, as described by the manufacturer’s protocol). The TRIAM concentration was chosen after titration experiments with propidium iodide (PI) staining to exclude toxicity. The H_2_O_2_ concentration was chosen after titration experiments with PI staining to generate a sub-maximal lethality rate. We were able to generate a sub-maximal MHCII expression after incubation with 100 U/ml IFN gamma [21]. To examine the MHCII expression after INF gamma stimulation with and without 10 μg/ml TRIAM treatment, we stained with MHCII Alexa 647 antibody and analyzed with flow cytometric analyses as described above.

### Tissue preparation, RNA isolation, and gene expression analyses with quantitative RT-PCR

Total RNA was isolated from the sciatic nerve samples of rats at disease maximum, 18 days p.i. using the RNeasy Mini extraction kit (Qiagen, Hilden, Germany). All samples were treated with the RNA Stabilization Reagent (RNAlater, Qiagen, Hilden, Germany) at 37 °C overnight and stored at − 80 °C until use. Total RNA was reverse-transcribed into cDNA according to the manufacturer’s protocol for the Reverse Transcription System (Promega, Madison, WI, USA).

Sequence-specific primers were designed with the following, sen (sense), ase (anti-sense), then mRNA expression levels were analyzed by quantitative RT-PCR according to the manufacturer’s instructions (Applied Biosystems, Foster City, CA, USA):

FoxP3 (sen AGG CAG AGG ACA CTC AAT GAA, ase ACT GCT CCC TTC TCA CTC TCC), IFN gamma (sen AAA GAC AAC CAG GCC ATC AG, ase CTT TTC CGC TTC CTT AGG CT), IL-10 (sen CCT GCT CTT ACT GGC TGG AG, ase TCT CCC AGG GAA TTC AAA TG), and IL-4 (sen TGA TGG GTC TCA GCC CCC ACC TTG C, ase CTT TCA GTG TTG TGA GCG TGG ACT C).

RT-PCR amplifications were carried out using the RT-PCR System 7500 (Applied Biosystems) via protocol described by Pfaffl et al. [22]. In this context, the relative expression ratio is calculated only from the RT-PCR efficiencies and the crossing point deviation of the sample versus a control. β-Actin and GAPDH were used to normalize relative mRNA expression. Each experiment was performed in duplicate, and the mean Ct was used in the equation for the housekeeping genes and Ct for the genes of interest.

Total mRNA was isolated from Schwann cells, and RT-PCR analyses were performed for TLR-4 (toll-like receptor-4, sense GCGCCTAAAACCCATTATGTT, anti-sense TGATTCTTTGCCTGAGTTGCT) and IL-1Ra (IL-1 receptor antagonist, sense GTGTGATGCCCCTAAACTGAA, anti-sense AACCTCTTTAGGCAGCTCTGG) as described above.

### Statistical methods

Statistical analyses were performed by GraphPad Prism 7 software (GraphPad Software Inc., San Diego, USA). Data are provided as mean ± SEM (standard error of mean) for the clinical score or as mean ± SD (standard deviation) for the rest of the statistical analyses. Differences between pairs of groups were tested by Student’s *t* test. Differences between three or more groups were tested by one-factor analysis of variance (ANOVA). The area under the curve (AUC) was calculated for clinical courses and analyzed by one-way analysis of variance (ANOVA) combined with Tukey’s multiple comparison test. The chi-square test was used to analyze categorical outcomes (disease incidence). Probability level (*p* value) are indicated as *p ≤ 0.05, **p ≤ 0.01, ***p ≤ 0.001, and ****p ≤ 0.0001.

## Results

### Intrathecal triamcinolone applied in a therapeutic concept ameliorates the clinical course of experimental autoimmune neuritis

**Fig. 1:**
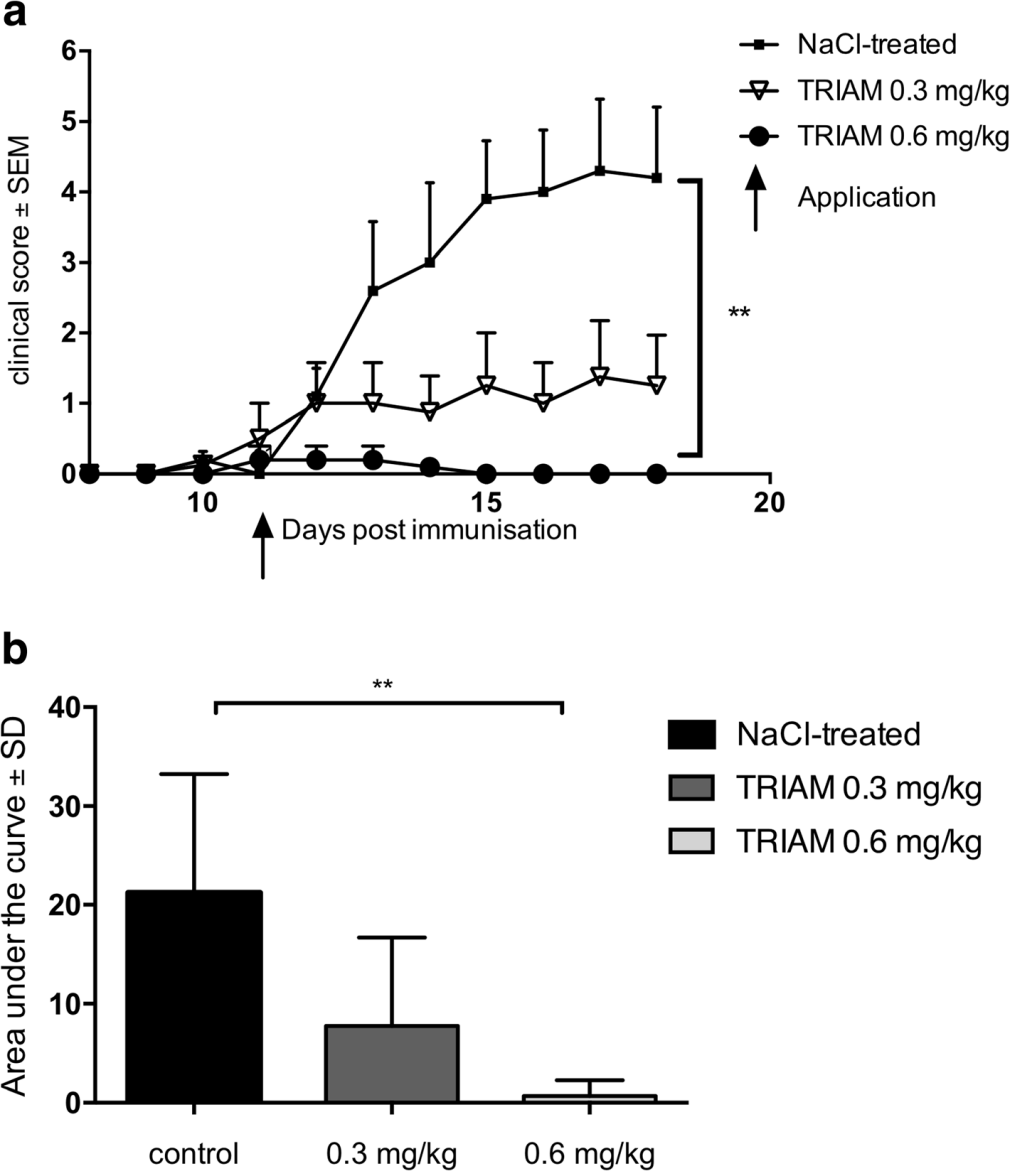
Representative clinical course of EAN rats. **a**. EAN was induced in Lewis rats by immunization on day 0 with P2_53–78_ peptide plus CFA. Rats received human triamcinolone (TRIAM) intrathecally at doses of 0.3 mg/kg and 0.6 mg/kg on day 11 p.i. Control rats received NaCl 0.9% only. The experiment was repeated two times, each time with *n* = 5/group. Mean values and SEM are depicted. Statistical analysis and *p* value calculation were performed calculating the area under the curve (AUC). **b** Comparison of AUC was done using one-way ANOVA combined with Tukey’s multiple comparisons test. Mean values and SD are depicted (NaCl-treated vs 0.6 mg/kg; **p < 0.001, *n* = 5)

After immunization with P2 protein peptide 53–78, clinical signs of EAN begun around day 10 p.i. At day 11 p. i., 0.9% NaCl or TRIAM were applied intrathecally. The incidence of EAN for the control group was 100%, and the groups receiving TRIAM showed an incidence of 50% (0.3 mg/kg) and 20% (0.6 mg/kg) respectively (**p < 0.01). A remarkable dose-dependent amelioration of EAN clinical signs was documented (AUC, NaCl-treated vs. 0.6 mg/kg TRIAM **p < 0.001, *n* = 5/group) (Fig. 1a, b).

### Intrathecal triamcinolone improved proximal and distal nerve conduction

To elucidate the effect of intrathecal TRIAM regarding proximal and distal demyelination, we performed electrophysiological measurements of the sciatic nerve at the maximum of the clinical course (day 18 p.i.) as described in the “Materials and methods” section.

The average minimum latencies of the elicitable F-waves were slightly prolonged in NaCl-treated group at day 18 p.i. compared to day − 1 p.i., whereas there was no difference for the 0.3 and 0.6 mg/kg TRIAM-treated groups (average minimum F-wave latencies of the control group on day − 1 p.i. 8.2 ms vs. day 18 p.i. 14.2 ms, *p < 0.05, *n* = 5, experiment repeated twice).

**Fig. 2:**
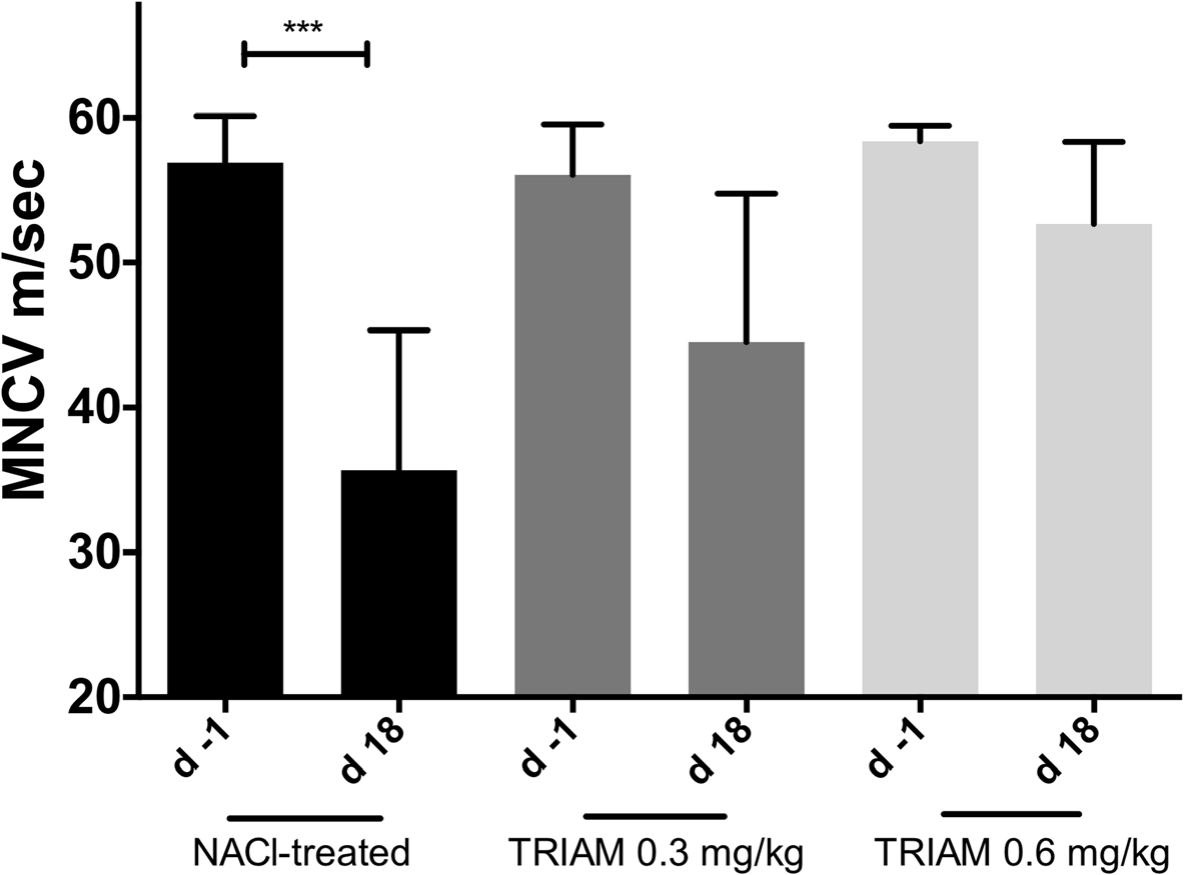
Motor nerve conduction velocity analyses of the sciatic nerve. Motor nerve conduction velocity of the sciatic nerve 1 day before immunization (d -1) and at the peak of disease (day 18 p.i.) (*n* = 5/group): After proximal and distal stimulation of the sciatic nerve, the conduction velocity (MNCV) was calculated. A statistically significant reduction of the MNCV appeared only for the NaCl-treated group (*n* = 5) but no difference in the MNCV was seen for all triamcinolone (TRIAM)-treated groups. Mean values and SD are depicted; *p* values, **p < 0.0001. The experiments were repeated two times with *n* = 5/group

MNCV was significantly reduced in the NaCl-treated rats (mean MNCV on day 18 p.i. 35.6 m/s vs. day − 1 p.i. 56.9 m/s, ***p < 0.0001, *n* = 5, experiment repeated twice), whereas no difference of the mean MNCV on day − 1 was seen for TRIAM-treated groups (Fig. 2), indicating the additional protective role of intrathecal application of TRIAM against distal demyelination.

### Intrathecal triamcinolone reduces histological signs of demyelination and T cell and macrophage infiltration in the sciatic nerves

**Fig. 3:**
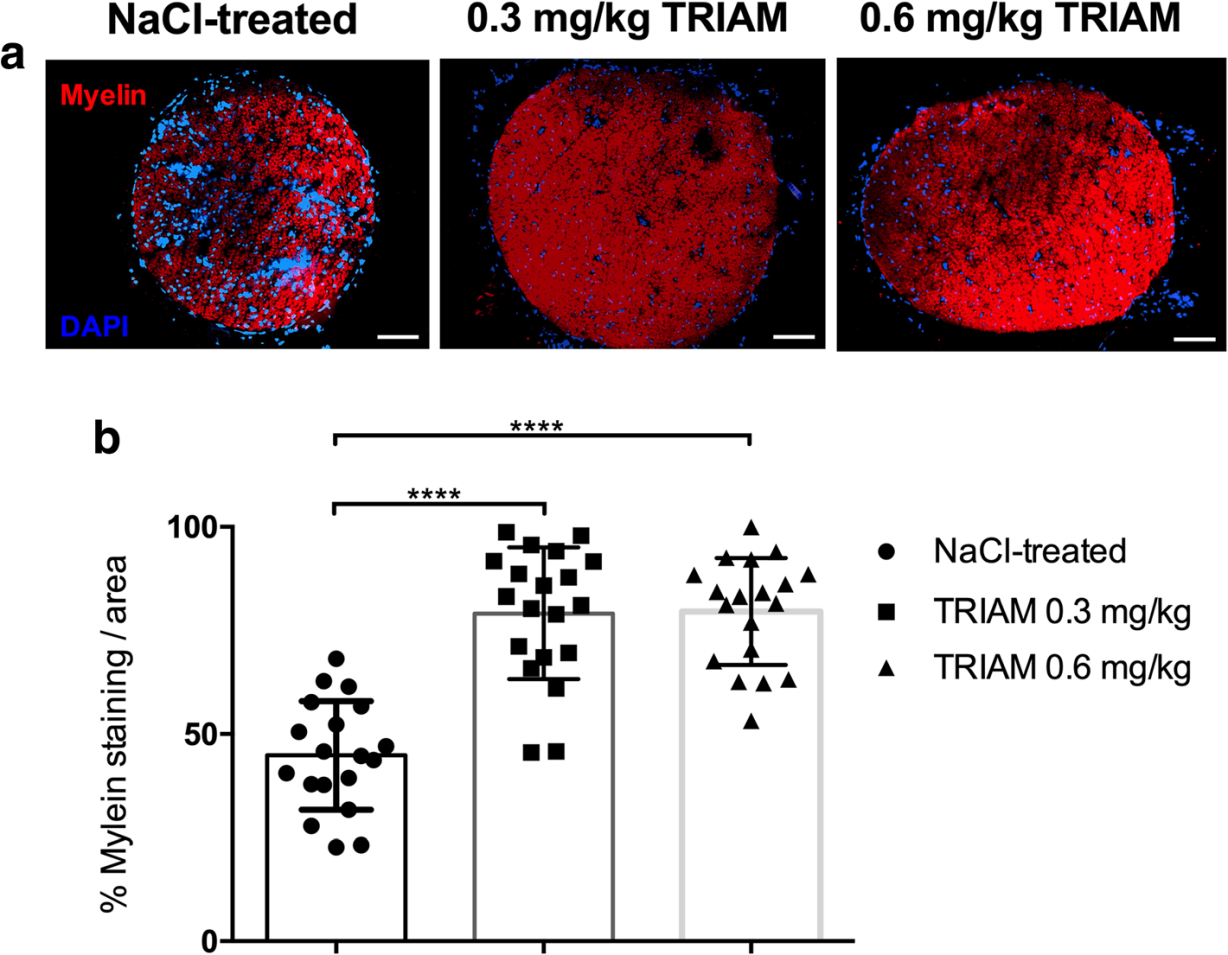
FluoroMyelin staining of the sciatic nerve. **a** Representative pictures and statistical analysis of FluoroMyelin staining for sciatic nerve transverse sections of rats (*n* = 5/group) treated with triamcinolone (TRIAM) 0.3 and 0.6 mg/kg and NaCl-treated animals, showing massive demyelination for NaCl-treated rats and preservation of myelin for triamcinolone-treated groups (**b**, mean values and SD are depicted, ***p < 0.0001) at day 23 p.i. No effect on demyelination was found on day 18 p.i. (experiments repeated twice). The experiment was performed once for day 23 p.i. Scale bars indicate 100 μm

Figure 3a shows representative pictures of FluoroMyelin™ staining. Administration of 0.3 and 0.6 mg/kg intrathecal TRIAM significantly reduced the percentage of demyelination of the sciatic nerves when compared to NaCl-treated rats on day 23 p.i. (***p < 0.0001, *n* = 5 Fig. 3b, experiment repeated once) but not on day 18 p.i. (experiment repeated twice, data not shown).

**Fig. 4:**
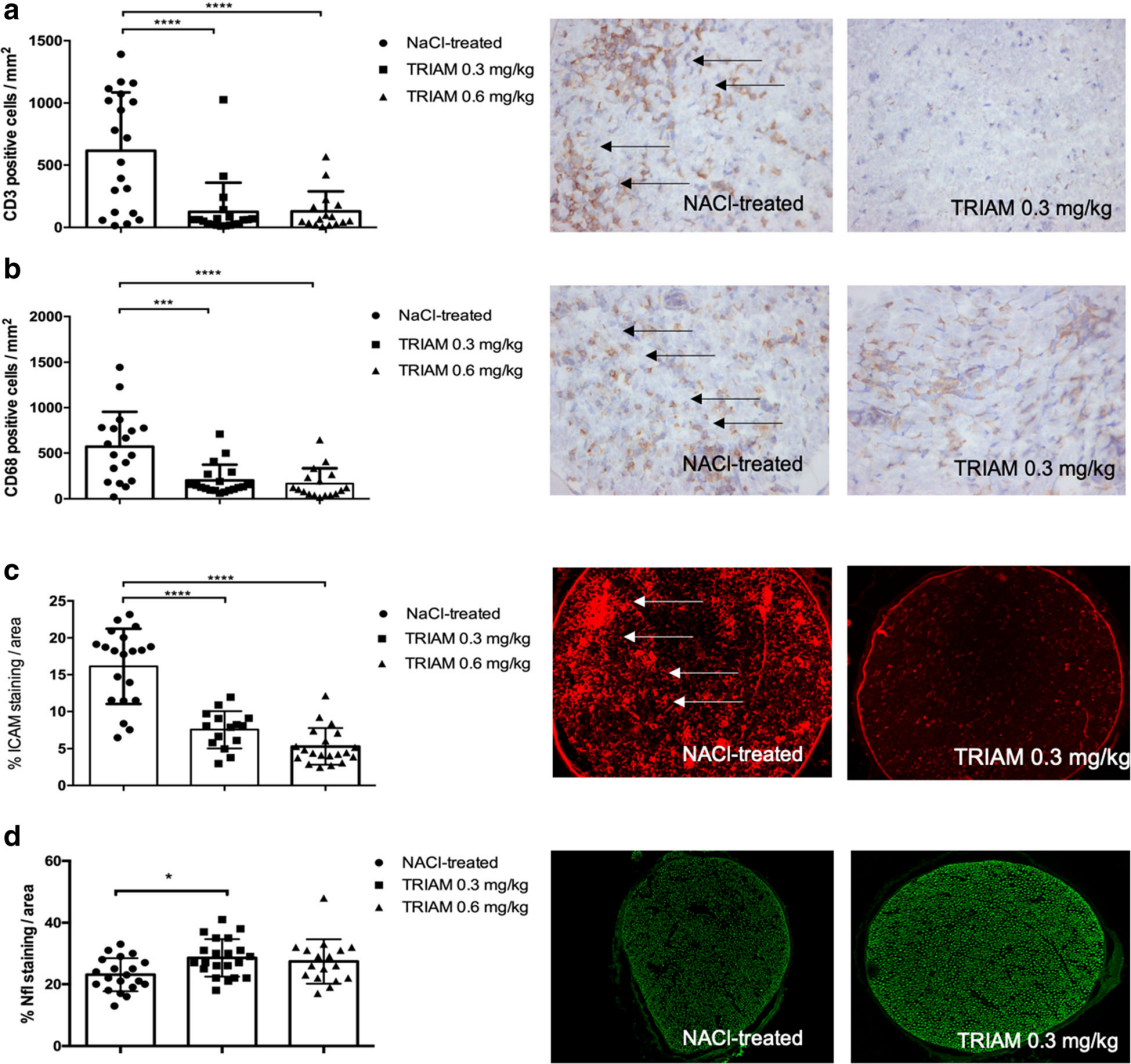
Histological analyses of the sciatic nerve. Histological analyses of the immune cell populations in the sciatic nerve, blood-nerve barrier permeability, and axonal staining with representative pictures for each staining. Scale bars indicate 100 μm for ICAM and neurofilament staining and 50 μm for the CD3^+^ and CD68^+^ staining. Mean numbers of **a** CD3^+^ T cells and **b** CD68^+^ macrophages per mm^2^ sciatic nerve sections as measured by immunohistochemistry on day 18 p.i. from EAN rats (*n* = 5/group) receiving intrathecal triamcinolone (TRIAM) in a therapeutic concept at different doses (0.3 mg/kg, 0.6 mg/kg) and NaCl-treated rats at day 11 post-immunization. Mean values and SD are depicted; ***p < 0.0001, *p < 0.05. The experiment was repeated twice with similar results. **c** Statistical analysis of ICAM staining for sciatic nerve transverse sections of rats (*n* = 5/group) treated with triamcinolone 0.3 and 0.6 mg/kg and NaCl-treated animals, showing a reduction of ICAM-1 histological expression for triamcinolone-treated rats, which implies a protective secondary effect on blood-nerve barrier permeability. Mean values and SD are depicted, ***p < 0.0001. The experiment was repeated twice with similar results. **d** Statistical analysis of neurofilament (Nfl) staining for sciatic nerve transverse sections of rats (*n* = 5/group) treated with triamcinolone 0.3 and 0.6 mg/kg and NaCl-treated animals, showing a preservation of axonal neurofilament staining for triamcinolone-treated rats, which implies a protective secondary effect on neuronal integrity. Mean values and SD are depicted, *p < 0.005

Next, we showed that clinical improvement correlates with the reduction of inflammatory infiltration of the PNS. Histological data demonstrating inflammation within the PNS are depicted in Fig. 4. Intrathecal administration of 0.3 and 0.6 mg/kg TRIAM on day 11 p.i. significantly reduced infiltration of macrophages and lymphocytes in the sciatic nerves compared to the NaCl-treated control group, when examined on day 18 p.i. (***p < 0.0001, Fig. 4a, b). Interestingly, inflammatory infiltrates were reduced both in the distal and proximal parts of the sciatic nerves (data not shown).

Furthermore, ICAM-1 expression was significantly reduced for 0.3 and 0.6 mg/kg-treated rats correlating with the prominent reduction of inflammatory infiltrates for these concentrations (***p < 0.0001, Fig. 4c). Axonal staining with neurofilament H antibody showed less axonal damage for the low TRIAM dosage (*p < 0.05, Fig. 4d).

### Intrathecal triamcinolone induced a Th2 cytokine shift in the sciatic nerve at disease maximum as well as a reduction of peripheral lymphocytes

**Fig. 5:**
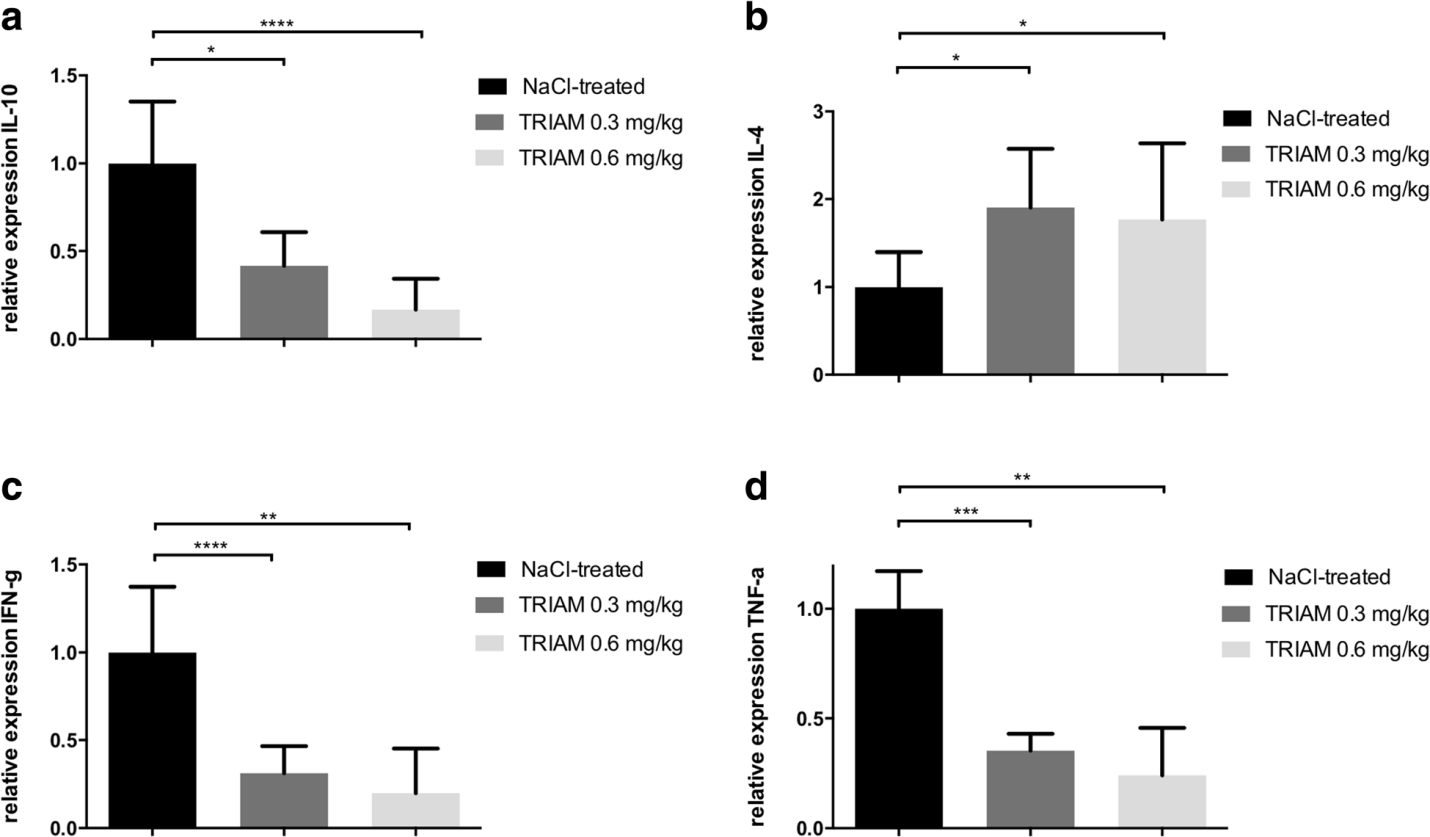
**a**–**d** RT-PCR analyses of the sciatic nerve. Relative expression of anti-inflammatory (IL-4 and IL-10) and proinflammatory (TNFα, IFN-γ) cytokines in the sciatic nerves at the peak of disease showing a Th2 anti-inflammatory cytokine shift after treatment with triamcinolone (TRIAM) 0.3 and 0.6 mg/kg compared to NaCl-treated animals. The experiment was repeated twice with similar results (*n* = 5/group). Mean values and SD are depicted

Furthermore, a Th2 cytokine shift was observed for both concentrations of 0.3 and 0.6 mg/kg TRIAM at day 18 p.i., as IL-4 mRNA expression showed a statistically significant increase in contrast to TNFα and IFN-γ, which decreased at the disease maximum. IL-10 however was also reduced at that time point (Fig. 5, ***p < 0.0001, *n* = 5/group, experiment repeated twice).

To detect possible effects on the secondary lymphoid organs, we also isolated peripheral mononuclear cells from the inguinal lymph nodes and spleen at day 18 p.i. and analyzed them with flow cytometry. We evaluated the frequency of CD4^+^ T cells, CD11b^+^ cells, CD4^+^ CD11b^+^ dendritic cells (DCs) and CD4+ CD25+ FoxP3+ regulatory T cells (Tregs). Apart from a reduction of the percentage of lymphocytes in spleen (lymphocytes in the control group; mean ± SD of 15.8 ± 4.4%, 0.3 mg/kg TRIAM 3.4 ± 0.3%, 0.6 mg/kg TRIAM 5.5 ± 1.6% of all cells, control group vs. 0.3 mg/kg group, **p < 0.001) no further statistically significant differences were found between TRIAM and NaCl-treated rats for all populations tested.

### Anti-inflammatory and antioxidative effects of triamcinolone on Schwann cell cultures

**Fig. 6:**
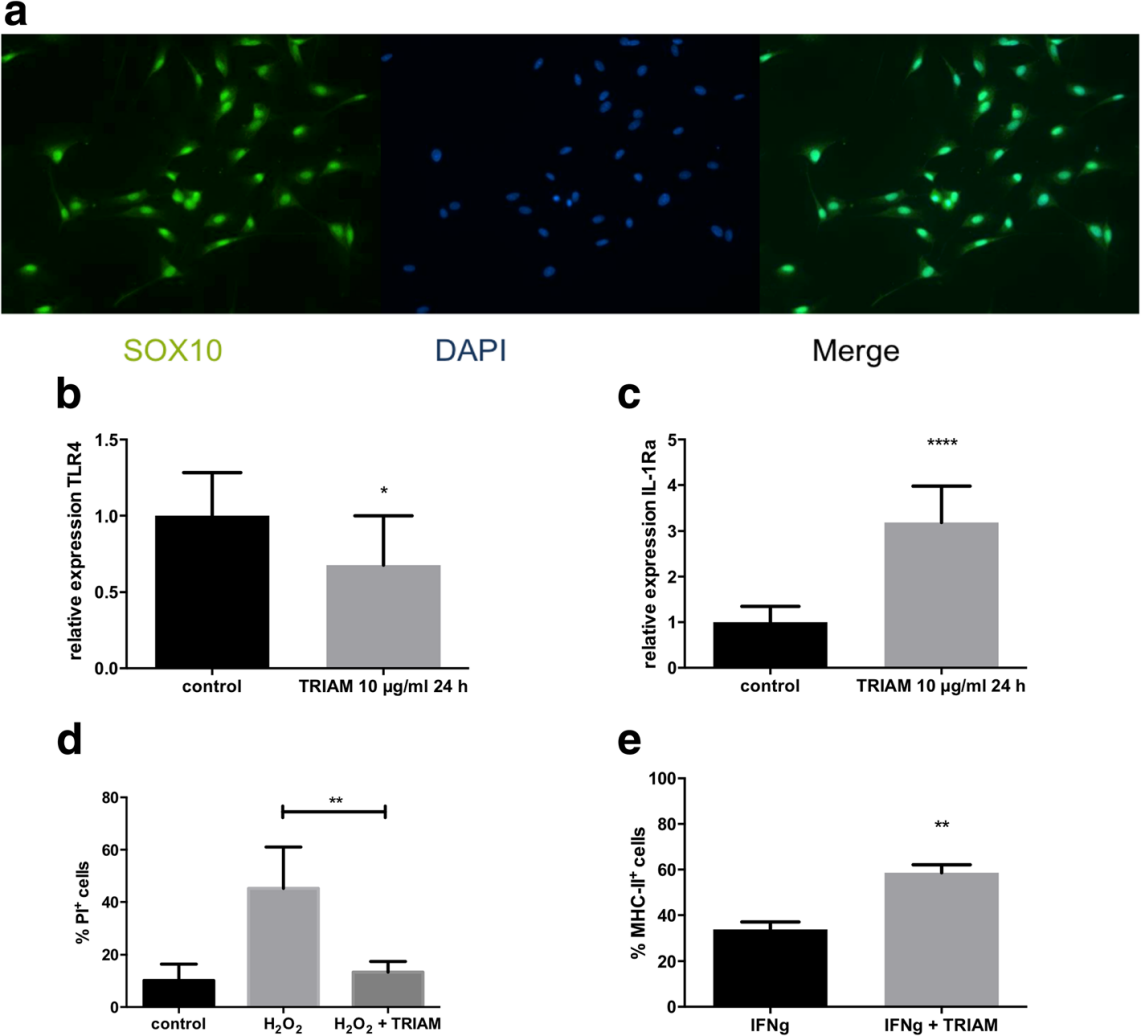
In vitro analyses of Schwann cells culture. **a** Immunohistochemical staining of Schwann cell culture with SOX10 and DAPI (nuclear staining). **b**, **c** After incubation of Schwann cells with 10 μg/ml triamcinolone (TRIAM) for 24 h, we detected a significant reduction in the relative gene expression of the pro-inflammatory TLR-4 as well as an increase in the anti-inflammatory cytokine IL-1Ra by RT-PCR. Mean values and SD are depicted. **d** Incubation of Schwann cells for 24 h with 0.03 mM H_2_O_2_. Co-incubation with 10 μg/ml triamcinolone resulted in increased cell viability in propidium iodide (PI) staining. Mean values and SD are depicted. **e** Incubation of Schwann cells with 100 U/ml interferon-γ. After co-incubation with triamcinolone, MHC II expression in flow cytometric analyses was significantly increased. Mean values and SD are depicted. *p ≤ 0.05, **p ≤ 0.01, ***p ≤ 0.001, and **** *p* ≤ 0.0001

Representative pictures of immunohistochemical staining of SC culture with SOX10 and DAPI are presented in Fig. 6a.

After incubation with 10 μg/ml TRIAM for 24 h, we were able to detect a significant reduction in the expression of the inflammatory TLR-4 as well as an increase in the anti-inflammatory cytokine IL-1Ra by RT-PCR (Fig. 6 b, c).

To induce oxidative stress, we incubated the SCs with 0.03 μM H_2_O_2_ for 24 h. Co-incubation with 10 μg/ml TRIAM resulted in an increased cell viability in PI staining (Fig. 6d).

To induce MHCII expression, we incubated SCs for 5 days with 100 U/ml interferon-γ. Interestingly, after co-incubation with TRIAM, MHCII expression was significantly increased. A potentially toxic effect of the substances used in the concentrations indicated was excluded by means of PI staining (Fig. 6e).

## Discussion

In our current study, we present a new perspective of the classical treatment of autoimmune neuropathies using the route of intrathecal application of TRIAM. Thereby, we introduce two novel aspects of the action of intrathecal TRIAM: an anti-inflammatory potential for autoimmune neuritis in vivo and a direct immunomodulatory/antioxidative effect on Schwann cells in vitro.

Intrathecal TRIAM has been established as an off-label treatment to improve spasticity and walking distance in patients with chronic forms of multiple sclerosis. Patients with spinal symptoms respond particularly well to this application route for up to 3 months after application. Due to the dose sparing effect, TRIAM therapy is rarely accompanied by long-term side effects, such as osteoporosis and weight gain, which are usually observed during repeated pulsed intravenous steroid infusions [4].

The mode of action of TRIAM is not completely understood since the scientific evidence of its efficacy relies on clinical observations and not on placebo-controlled trials. According to studies on MS patients, a potential effect of intrathecal TRIAM characterized in CSF is the reduction of repulsive guidance molecule A (RGMa), a cell death regulator [23]. Recurrent TRIAM applications induced a decreased concentration of RGMa fragments combined with a decline in free radical concentration, potentially improving neuronal regeneration [6, 7].

On the other hand, there is only one study suggesting that TRIAM could be effective in the treatment of peripheral neuropathies and most specifically on the treatment of neuropathic pain. TRIAM was applied subcutaneously 5 days after a nerve injury to rats with an experimental post-traumatic painful peripheral neuropathy. TRIAM-treated rats had a statistically significant reduction in the magnitude of heat-hyperalgesia and mechano-allodynia. The proposed mechanism of action was a reduction of TNFα in endoneurial mast cells [24].

However, the mode of action of the intrathecal application has not been investigated for autoimmune diseases of the PNS despite proximal route involvement and early intrathecal protein increase.

We show for the first time, that in a therapeutic concept, TRIAM achieved a dose-dependent improvement of clinical signs, in combination with a remarkable reduction of inflammatory infiltrates in the sciatic nerves. Nerve conduction studies showed a prominent preservation of F-waves as an indicator of an improvement of proximal demyelination as well as an unchanged motor conduction velocity as a sign of a distal preservation of myelination. Demyelination reduction was more pronounced at day 23 p.i. and not at day 18 p.i. (maximum of disease) implying a late effect of the reduction of the inflammatory infiltrations on demyelination or a late improvement of remyelination. Therefore, we conclude that intrathecal TRIAM injection in the effector phase of autoimmune neuritis reduces inflammatory activity beginning at the injection sites and extending into the whole nerve thereby achieving enough improvement of electrophysiological signs of demyelination.

To define possible mechanisms of action of the local intrathecal injection of TRIAM, we analyzed possible immunomodulatory mechanisms in the PNS reported before for systemically applied corticosteroids.

Firstly, the reduction of inflammatory infiltrates correlated with the downregulation in the histological expression of ICAM molecule in the peripheral nerves. This effect has been reported before for patients with chronic inflammatory demyelinating neuropathy and is probably secondary to the reduction of mononuclear cell infiltration [25]. This observation is crucial and could be easily evaluated in patients with CIDP receiving repeated intrathecal TRIAM applications, through a reduction of total protein in the CSF, which is the marker of blood-nerve barrier permeability. Neurofilament staining showed a reduction of axonal loss after TRIAM treatment probably as a secondary effect to the impressive anti-inflammatory effect of TRIAM.

Furthermore, the anti-inflammatory effects on cell infiltrates correlated with a Th2-cytokine (anti-inflammatory) shift (increase of IL-4 and decrease of IFN-γ and TNFα) in the peripheral nerves, confirming the effects of TRIAM in the gene expression level. Our findings are crucial as previous experiments have shown that activated glucocorticoid-receptor complex can bind to and inactivate key proinflammatory transcription factors (e.g., NF kappa B). While steroid treatment broadly attenuates cytokine production, it cannot modulate it selectively, e.g., just the Th0, the Th1, or the Th2 pathways. The production of the “anti-inflammatory” IL-10 is also inhibited, as shown in our experiments [26]. However, through an increase of IL-4, a Th2 cytokine shift is still achieved in our model, which implies an effective anti-inflammatory action.

We next considered the potential site of action of intrathecal TRIAM in the PNS, focusing on SCs, as the major cell population initially affected during demyelinating neuropathies. Previous in vitro studies reveal that SCs express glucocorticoid receptors whereas GR is expressed in the nuclei of SCs in vivo in intact and injured sciatic nerves. Glucocorticoids (hydrocortisone, dexamethasone) enhance the potency of SC proliferation [27, 28]. Furthermore, they enhance the rate of myelin formation [29] and stimulate the activity of promoters of peripheral myelin protein-22 (PMP22) and myelin protein zero (P0) genes [30, 31].

However, the in vitro effects of TRIAM on SCs have not been investigated before. Our data imply a combined anti-inflammatory and anti-oxidative effect of TRIAM on SCs.

Lipopolysaccharide-induced inflammatory cytokine production by SCs is dependent upon TLR4 expression. In vitro data showed that TLR4 expression is upregulated after sciatic nerve injury of rat and modulation of its expression increased relative gene expression of proinflammatory molecules such as c-Jun and extracellular signal-regulated kinase (ERK) [32]. In our in vitro model, TRIAM reduced significantly TLR4 expression implying an anti-inflammatory potential of SCs after TRIAM treatment.

Furthermore, IL-1Ra production was reduced after SC incubation with TRIAM. Experiments on sciatic nerves of EAN rats after immunization with P2 and after adoptive transfer of effector T cells have shown that in both models, IL-1a was expressed by SCs, during preclinical EAN whereas IL-1Ra was not detectable in SCs at this stage. However, clinically manifest EAN was characterized by SC-specific expression of IL-1Ra, mostly on the paranodal regions, sites essential for proper impulse transmission. These data indicate that SC-specific autoregulation of IL-1Ra is highly relevant for immune regulation at paranodes during autoimmune neuritis [33].

TRIAM also protected SCs from H_2_O_2_-mediated oxidative stress. Oxidative stress is the main characteristic of autoimmune inflammation and leads to neuronal degeneration, a crucial parameter of disability for patients with chronic immune neuropathies. Treatment options, which reduce oxidative stress and therefore improve neuronal survival, are therefore essential for these patients. Furthermore, in line with our experiments, CSF analyses in MS patients (*n* = 16) after one intrathecal TRIAM injection revealed an increase of Cu (II) ion absorption, which reflects an augmented content of reduced proteins and an alteration of the redox potential in cerebrospinal fluid, through a decline of reactive oxygen species [34].

SC showed however a pro-inflammatory phenotype after TRIAM and IFN-γ incubation through the increase of MHCII expression. We interpret these results in the context of the crystalline structure of TRIAM, which could be presented as antigen on MHCII class molecules thereby initiating an immune response. This effect could explain cases of constrictive arachnoiditis and sterile meningitis reported after repeated intrathecal TRIAM injection [4]. However, we must point out that CSF investigations regarding markers for cell damage, such as neuron-specific enolase, S-100, neurofilament heavy chain, tau protein provided no convincing evidence of neuronal or SC damage after the intrathecal TRIAM treatment [35]. The interaction of substances applied intrathecally with the MHCII molecule and its role for sterile meningitis remains to be further investigated as other substances with non-crystalline structure can also lead to this side effect [36]. As we did not investigate the effects of TRIAM in neuron-SC cocultures regarding myelination, we can only postulate that the late effect on myelination shown on day 23 p.i. could be attributed on an improved SC myelination function.

## Conclusions

In conclusion, we present a new perspective for corticosteroids treatment in peripheral neuropathies by applying intrathecal TRIAM in a therapeutic concept.

The major shortcoming of our in vivo study is the fact that the monophasic animal model of EAN does not reproduce the complexity of the pathophysiology of CIDP but mostly the one of GBS, for which at least intravenous corticosteroids are not effective. Therefore, we chose the therapeutic concept in order to investigate the effects of TRIAM in the effector phase (when clinical signs begin to appear), which can be recognized both in GBS and CIDP patients in everyday practice.

Since intrathecal TRIAM treatment has already been used for patients with multiple sclerosis, the transfer of our application model in human subjects with CIDP could be tested in clinical studies. As corticosteroids are already used for patients with CIDP in the case of sensory symptoms and painful neuropathic pain, a probable anti-inflammatory, anti-oxidative, and secondary neuroprotective beneficial effect of intrathecal TRIAM application as well the dose-sparing effect must be considered. In light of our current observation, intrathecal application of TRIAM could be a promising treatment option for neuritis patients.

## Abbreviations

AUC: Area under the curve
CFA: Complete Freund’s adjuvant
CIDP: Chronic inflammatory demyelinating polyneuropathy
CMAP: Compound muscle potentials
DAPI: 4′,6-Diamidino-2-phenylindole
EAN: Experimental autoimmune neuritis
GBS: Guillain-Barré syndrome
i.p.: Intraperitoneally
ICAM1: Intercellular adhesion molecule 1
MNCV: Motor nerve conduction velocity
p.i.: Post-immunization
PBS: Phosphate-buffered saline
PNS: Peripheral nervous system
TRIAM: Triamcinolone acetonide
